# Factors that affect the implementation of an integrated care programme for older people with different frailty levels: a qualitative study of commissioners and provider stakeholders

**DOI:** 10.1186/s12877-024-05412-4

**Published:** 2024-10-14

**Authors:** Nimra Khan, David Hewson, Gurch Randhawa

**Affiliations:** 1 Department of Psychiatry, University of Oxford, Warneford Hospital, Warneford Ln, Headington, Oxford, OX3 7JX UK; 2 Institute for Health Research, University of Bedfordshire, Hitchin Road, Luton, LU2 8LE UK

**Keywords:** Integrated care, Older people with different frailty levels, Frailty, England

## Abstract

**Introduction:**

The NHS has made it mandatory for General Practices in England to proactively identify and manage older people with moderate and severe frailty since the GMS contract of 2017/2018. In Luton, stakeholders developed the Luton Framework of Frailty (LFF) to implement this national policy. The aim of this study was to explore the factors that affect the implementation of this national policy at a local level.

**Methods:**

In-depth interviews were conducted with 18 commissioners and service providers, all of whom were involved in providing services for older people with different frailty levels (OPDFL). Purposive and snowball sampling methods were used, with thematic analysis used for data analysis.

**Results:**

Two main themes with several sub-themes were found. The first theme was the tension within existing national policy initiatives to provide integrated care services for OPDFL, which illuminated their strengths and limitations. Participants felt that new initiatives, such as the development of Primary Care Networks and Enhanced Health in Care Homes, have improved primary care coordination. However, the traditional reactive approach for managing older people who are frail was thought to be counterproductive, when an approach that focused on prevention and early intervention would have been better. The second theme concerned the contextual factors that affect implementation of integrated care. These included having key leaders at a local level, the requirement for more funding, as well as the need for good working relationships among service providers. However, the lack of awareness about the care pathways among GPs was thought to be a reason for the variation in the implementation of the LFF. The COVID-19 pandemic was perceived as a challenge for the implementation of the LFF. Finally, polices were thought to succeed only if more resources are provided, while the term frailty should be used with caution due to the negative connotations of OPDFL towards this term.

**Conclusion:**

The implementation of an integrated care programme for OPDFL can be affected by several factors. Having proactive national policies that facilitate coordination and, having key leaders locally, the need for more funding, and good working relationships, are some of the contextual factors that could facilitate a successful implementation. In contrast, the lack of awareness of the care pathways that have been introduced locally, insufficient resources to deliver the programmes efficiently and a lack of careful consideration of how the term frailty is used could hinder this being put into practice.

## Introduction

In the UK, the proportion of older people is increasing, with one in four people predicted to be over the age of 65 by 2050 [1]. Although this increase in longevity is a success story of public health, an increased older population could put strain on both health and social care systems [2]. One reason for this strain is the increased prevalence of age-related conditions, such as frailty [3–5]. Compared to older people who are robust, those with frailty are more likely to experience adverse outcomes such as falls and fractures, institutionalisation, ambulance and emergency visits, hospital admissions and death [6]. This in turn poses a huge burden to older people, their informal caregivers, as well as to the health and social services, which already have limited resources [7].

**Fig. 1:**
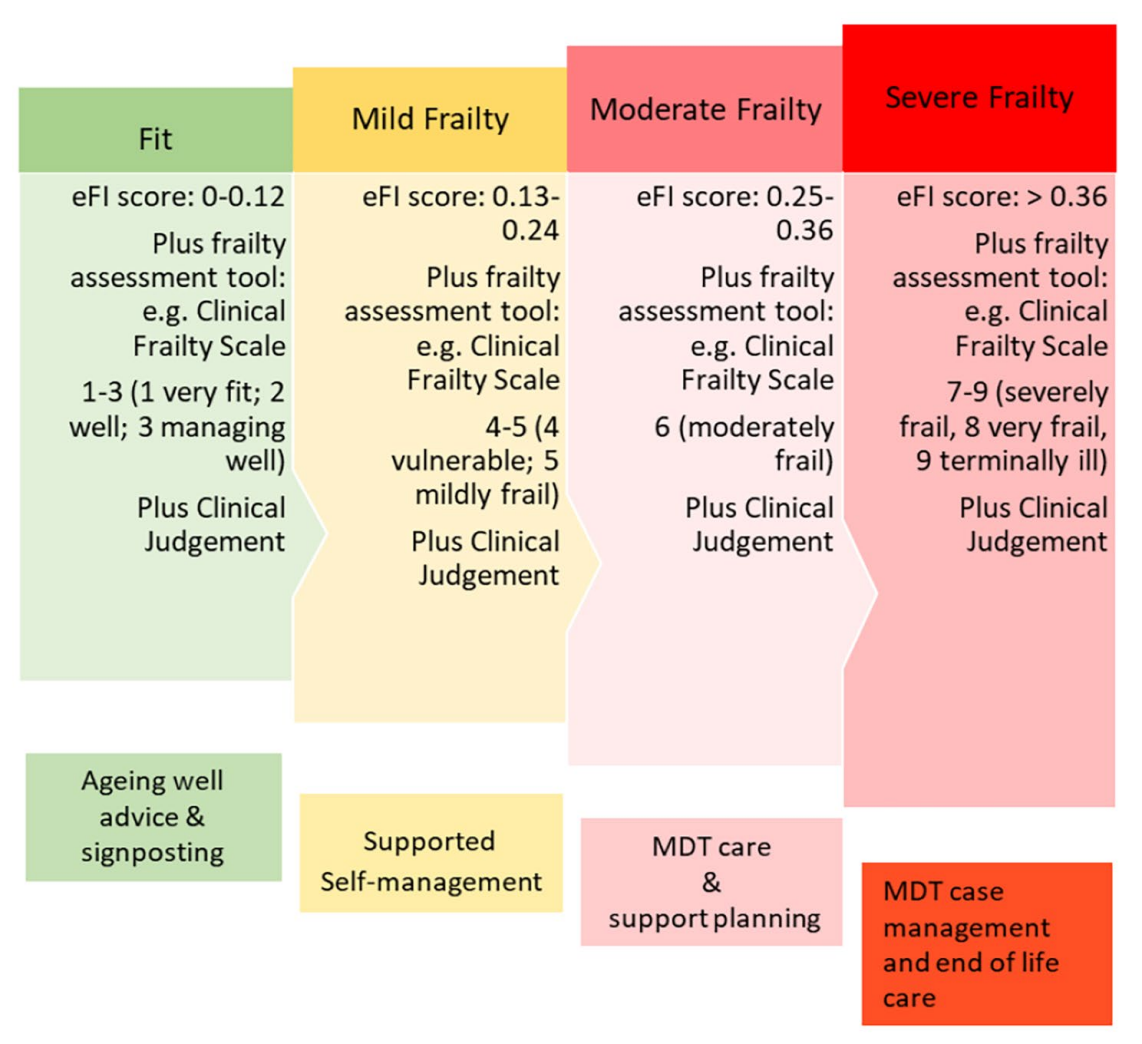
Luton Framework for Frailty

Frailty is one of the biggest challenges facing the economically stressed National Health Service (NHS) of the UK. Primary prevention has been proposed as the main strategy to reduce the costs of frailty management [7, 8]. The primary care providers are expected to proactively plan and manage care for older adults with frailty to ensure continuity of their care and to promote improved outcomes. This commitment to improving the quality of care for older people is demonstrated by the introduction of the requirement in the General Medical Service (GMS) contract in 2017/2018 for general practices in England to proactively identify and manage all older people (≥ 65 years) with moderate or severe frailty. These policy recommendations have been used by the Luton Clinical Commissioning Group (CCG) to develop the Luton Framework of Frailty (LFF), which is presented in Fig. 1. The LFF is an integrated programme delivered to older people (≥ 65 years) who are residents of Luton [8].

At the general practice level, an older person (aged ≥ 65 years) is screened for their level of frailty using the electronic frailty index (eFI) [9]. The eFI has been developed based on the accumulation of deficits model, which classifies frailty levels based on the sum of the deficits present in an individual. The eFI calculates the sum of deficits from electronic health records (EHR) collected routinely in primary care, with deficits including abnormal test results, clinical signs, symptoms, diseases and disabilities [10]. The eFI, which consists of 36 deficits, was validated in a study using 900,000 EHR, and has been shown to strongly predict adverse outcomes [9, 11]. The eFI categorises frailty as robust (0 to 0.12), mild (> 0.12 to 0.24), moderate (> 0.24 to 0.36) and severe (> 0.36), based on the fraction of deficits present. The eFI score needs to be confirmed by clinical judgement.

In the LFF, an appropriate intervention is offered based on the person’s frailty level. For instance, those people who are categorised as fit are sent a leaflet each year on how to age well, with information provided on how to stay healthy. People who have mild frailty are offered a free 12-week exercise programme, which is called the “Healthy Ageing Programme”. Finally, for those people who are identified as moderately or severely frail, a case management evaluation by a multi-disciplinary team (MDT) is provided. In this latter case, the MDT coordinates with the older person and their family to develop a personalised care plan.

In addition, the Government has introduced several other initiatives to improve coordination, such as the development of Primary Care Networks (PCNs). This is a structural arrangement to bring together GP practices in the same area to address disease prevention and management [12]. Another initiative is the Enhanced Health in Care Homes (EHCH), which requires a care home to be aligned with a named PCN, which should improve the coordination between general practice and care homes. This should result in people living care homes receiving the same level of care that they would have received if they were still living in their own home [13].

The GMS contract had been in place for four years at the time of this study, but it was unknown how effectively the national policy has been translated at a local level. Therefore, the aim of this study was to evaluate the impact of the national policy at a local level by identifying factors that affect the implementation of the Luton Framework for Frailty.

## Methods

The aim of this study was to explore the factors that affect the implementation of an integrated care programme, the LFF. The study was conducted in Luton, which is a town in the southeast of England, which has a population of 225,300, with 11.7% of people aged over 65 years [14]. Luton is a diverse town, with over half of its population belonging to minority ethnic groups and an estimated 150 languages and dialects spoken. Even prior to the UK’s cost of living crisis in 2022/23, Luton had a high poverty rate, particularly among children and older individuals. However, poverty rates vary widely across Luton, with some wards, such as Biscot and Dallow, having a high percentage of older adults living in poverty of over 40%, compared to less than 8% other wards of Luton, such as Bramingham. This could be due to the intersection between race and frailty, which has put an increased burden on people from non-white backgrounds, who often have poor healthcare experiences and postpone care-seeking [15]. Furthermore, people from minority ethnic groups are more likely to be frail compared to their non-white counterparts, with frailty starting at an earlier age for them [16, 17]. In some cultures, frailty is considered a natural consequence of ageing, which would lead people to avoid seeking help, therefore, interventions should be designed while taking cultural nuances into account.

The Luton CCG helped to identify stakeholders across the system who played a key role in the design and delivery of the LFF. Participants were from both statutory and non-statutory organisations, with inclusion criteria consisting of their strategic engagement in planning and managing care processes, their involvement in implementing the LFF programme, and finally that they were representative of the diverse stakeholders and care providers involved in the care of OPDFL. This included people in senior management roles, commissioners, as well as healthcare professionals such as GPs, nurses, and pharmacists, while care coordinators were also included.

The participants were recruited using purposive sampling, with recruitment occurring from November 2020 to March 2021. This is a useful method of recruitment when participants need to meet specific inclusion criteria. Furthermore, since it was difficult to recruit certain health professionals such as GPs, snowball sampling techniques were also used by asking GPs known to the researcher if they could help to recruit additional participants. A participant information sheet was shared, and participants were requested to respond to the email with their decision as to whether they would participate in the study or not within two weeks. If they agreed to participate, an informed consent form was shared via email. Furthermore, due to COVID-19, face-to-face interviews were not possible, so participants were offered online data collection using the platform of their choice, such as Skype, Zoom or Microsoft Teams.

This study used semi-structured interviews to capture data from commissioners and service providers involved in providing services to OPDFL in Luton. A comprehensive topic guide was developed based on an extensive review of relevant literature, with this literature searching using a range of databases, including Web of Science and PubMed, with key terms used such as ‘frailty’, ‘older people’, and ‘integrated care’. In addition, a Google Scholar search was also conducted, while the reference lists of articles found were also examined to ensure all relevant materials were considered.

The interview guide was then pilot-tested with several service providers from different areas in England. These included two GPs based in London who had experience of working with older people with frailty, a nurse based in Luton, and a registrar at a hospital in Dudley. The final set of questions were then related to the barriers and facilitators faced when implementing the LFF. Data were analysed using thematic analysis, which is an interpretive approach of identifying patterns in data to explain a phenomenon [18]. The present study employed Braun and Clarke’s (2006) six-step approach to thematic analysis. Firstly, transcripts were read and reread, with detailed notes taken to capture the initial impressions of the data. Open coding was then conducted to identify patterns in the data that were pertinent to the research question. While the data analysis was primarily inductive, the data collection was informed by a topic guide, which resulted in some predetermined codes, such as exploring the barriers and facilitators to the implementation of the program. These codes were carefully examined, with themes identified by grouping together those codes that were related. The themes were subsequently reviewed, with all the relevant data read again to assess the extent to which it aligned with the themes, and to evaluate the interrelationships among the themes. This process of thematic analysis was iterative, with regular meetings held within the team to discuss the findings and refine the analysis. NVivo software was used for data analysis (Lumivero, Denver, CO, USA). Ethical approval was obtained from the Ethics Committee of the Institute for Health Research, University of Bedfordshire (IHREC950).

## Results

Data collection took place during the initial two waves of the COVID-19 pandemic, which presented challenges in terms of recruiting healthcare professionals, due to their busy pandemic management responsibilities. In total 22 service providers and commissioners were approached, of which 18 responded and took part in the study. Primary care professionals were adequately represented in the study, with data saturation achieved after the first 13 interviews. However, despite several efforts, the participation of secondary care professionals remained limited, as shown in Table 1, which contains participants’ job descriptions.

**Table 1:** Job descriptions of the participants

Participant	Job Description
P1	Senior Leadership Role (Primary care, strategic role)
P2	Senior GP (Primary care, strategic role)
P3	Senior Manager of a Service (Specialist service, strategic role)
P4	Senior Manager of a Service (Specialist service, managerial and frontline care role)
P5	Pharmacist (Primary care)
P6	Senior Manager of a Service (Specialist service, managerial role)
P7	Senior Geriatrician (Secondary care, strategic and frontline care role)
P8	Team Lead for a Service (Specialist service, managerial role)
P9	Team Lead for a Service (Specialist service, managerial role)
P10	Senior Leadership Role (Voluntary sector, strategic role)
P11	Senior Pharmacist (Primary care)
P12	Pharmacist (Primary care)
P13	Commissioner (Primary care)
P14	Senior Commissioner (Primary care, strategic role)
P15	GP (Primary care)
P16	Senior GP (Primary care, strategic and frontline care role)
P17	GP (Primary care, frontline care role)
P18	Senior GP (Primary care, strategic and frontline care role)

The thematic analysis identified two main themes, each of which had several sub-themes (Table 2).

**Table 2:** Themes and sub-themes

Theme 1: Existing national policy initiatives to provide integrated care services for OPDFL	Theme 2: Contextual factors that affect the implementation of an integrated care programme for OPDFL
Subtheme 1 A: National initiatives to improve primary care coordination.	Subtheme 2 A: Key leaders play important roles in promoting the uptake of the programme.
Subtheme 1B: The reactive approach has traditionally been prioritised over preventative measures	Subtheme 2B: Availability of funding
	Subtheme 2 C: How engagement and communication affect the delivery of an integrated care programme for OPDFL
	Subtheme 2D: Organisational restructuring often diverts professionals’ attention from present projects
	Subtheme 2E: Variation in implementation of the Luton framework for frailty
	Subtheme 2 F: The COVID Pandemic stopped preventative care pathways for OPDFL.
	Subtheme 2G: Local capacity is not enough to deliver the national policy of identifying and managing all older people with moderate and severe frailty
	Subtheme 2 H: Older people do not want to be labelled as ‘frail’

### Theme 1: National policy initiatives adopted to provide integrated care services for OPDFL

#### Subtheme 1 A: National initiatives to improve primary care coordination

Stakeholders talked about how national initiatives have improved coordination at the primary care level, such as the development of primary care networks (PCN) through which practices are grouped to form networks. The enhanced health in care homes (EHCH was also mentioned, with this initiative ensuring the alignment of care homes to a PCN.

*“The way the practices have been divided into networks…so it means that it is easy to coordinate general practice…and the other thing the network has done is align a care home to a network…so that’s been helpful …”* (P2, Senior GP).

*“I think as well*,* people were really committed in terms of frailty. I suppose nationally*,* was a bit of a hot topic…” (P9*,* Team Lead for a Service).*

#### Subtheme 1B: The reactive approach has traditionally been prioritised over preventative measures

Some participants perceived that there has been a focus on patients who have more complex needs, and that there are fewer preventative measures for older people who are fit. They also talked about how traditionally the focus has been on the prevention of hospital and nursing home admissions, with less focus on a person’s quality of life.

*“…we focus too much on those who’ve got complexity and frailty and we too often forget to do the preventative work for those who are functioning well at the moment…the other thing is and that continues to be a challenge*,* is we focus on prevention of admissions to hospitals and care homes when in fact what we should really be focusing on is people…” (P16*,* Senior GP).*

*“I mean*,* for me*,* it’s about prevention too to try and enable people to live as independently as possible. It’s a challenge*,* I think*,* between identifying frailty and labelling people as being sick and frail*,* as opposed to supporting their independence” (P17*,* GP).*

### Theme 2: Contextual factors that affect the implementation of an integrated care programme for OPDFL

#### Subtheme 2 A: Key leaders play an important role in promoting the uptake of the programme

Most of the participants named some key individuals in leadership roles, who they believed played an important role in encouraging the engagement of stakeholders locally with the programme.

*“So that was a really key factor in terms of having that senior sponsorship*,* and someone that was respected in the system” (P14*,* Senior Commissioner).*

*“We had a really excellent program lead. Having somebody whose focus of work was on this ….and could help us break it down and do bite-sized chunks of work and move it forward” (P16*,* Senior GP).*

#### Subtheme 2B: Availability of funding

Several participants talked about identifying frailty as a local priority area and allocating more funding as a factor that supported frailty work.

*“Frailty was identified as a priority and then more resource was given to it” (P13*,* Commissioner).*

*“Primary care incentive scheme incentivise practices to complete frailty reviews” (P15*,* GP).*

#### Subtheme 2 C: How engagement and communication affect the delivery of an integrated care programme for OPDFL

Many felt that the good working relationships between partners from different organisations were helpful. Some participants talked about the real will that all of the stakeholders had to do things differently. There was also an awareness across the system that they could not continue the way they had always been, and there was a need to improve services for older people.

*“We had good working relationships across our community services and secondary care services…we had meetings*,* where GPs met with a consultant from the hospital*,* that enabled the challenges to be discussed and agreed [on the] approach to support patients. Secondary care understood the challenges from a primary care perspective and primary care understood the challenges from [a] secondary care perspective” (P13*,* Commissioner).*

*“There was an appetite in the system to make a difference*,* and those stakeholders you know voluntarily came forward…to map what was currently happening and to identify the gaps and together design what it should look like so there was real co-production…” (P14*,* Senior Commissioner).*

Nevertheless, some participants felt that there is less system-level awareness of the existing care pathways and referral mechanisms, and it becomes especially challenging when people are on different information systems. They also mentioned that if a population cohort is not among the focus area of the PCN then they are less aware of the initiatives taken related to this population.

*“As doctors you’re so busy firefighting appointment after appointment; you’re not always aware of what’s happening…” (P18*,* GP).*

*“If a population group isn’t a priority focus for a GP practice. Then there’s less awareness…” (P14*,* Senior Commissioner).*

*“I think the other big barrier is dealing with social services because they’re on a completely different system… This happened the other day when I was trying to arrange an urgent occupational therapy assessment…I spent 35 minutes on the phone and they said*,* ‘oh*,* can you just send an email’*,* and then I sent an urgent email but I don’t know whether they looked at that email or it’s been actioned…” (P18*,* GP).*

#### Subtheme 2D: Organisational restructuring often diverts professionals’ attention from present projects

Participants mentioned that the recent change introduced in governance structures, whereby the three CCGs of Bedfordshire (Bedfordshire Clinical Commissioning Group, Luton Clinical Commissioning Group and Milton Keynes Clinical Commissioning Group) were being merged to form one. Participants talked about the kind of impact it could have, such as staff lacking local knowledge about other areas, and fewer people being used to deliver the programme over a wider area. They also mentioned that professionals who have been working on the LFF have been redeployed to new roles, and that organisational changes often result in people forgetting about ongoing projects.

*“… three CCGs in Bedfordshire are now merged to become one CCG…And the risk with doing all of those changes is that the projects you’ve got going potentially get slightly lost” (P2*,* GP).*

*“…we have got a Healthy Ageing Project in place but it is not in place in Bedford and Milton Keynes so we’ve got to think about how we will implement it there… We are reducing the CCG workforce to stop duplication but at the same time there would now be fewer people to deliver in a wider area and I think it will also lead to lack of local knowledge…” (P13*,* Commissioner).*

#### Subtheme 2E: Variation in implementation of the Luton framework for frailty

One participant felt that there is a variation in the way the programme is implemented across Luton, mainly because PCN prioritise different populations.

*“Different Primary Care Networks have different priority population groups*,* so they are at different stages of implementing the framework” (P6*,* Senior Manager of a service).*

#### Subtheme 2 F: COVID Pandemic stopped the preventative care pathways for OPDFL

Many participants talked about COVID, specifically how it had stopped all of the proactive frailty work, while they also mentioned that face-to-face appointments are better than virtual ones, especially for the older population.

*“Covid has made everything become reactive…Covid in general has meant that …. the proactive work has been put on hold” (P2*,* Senior GP).*

*“so a lot more is done by telephone and I think that is not necessarily the best way for some of the older population*,* because their hearing*,* and the way that you can’t pick up those cues and*,* you know*,* certainly discussing things like what you want for your future is very difficult” (P4*,* Senior manager for a service).*

#### Subtheme 2G: Local capacity is not enough to deliver the national policy of identifying and managing all older people with moderate and severe frailty

Most of the participants felt that the framework identifies a huge number of people with moderate or severe frailty, which means that with current staffing levels, it is not always possible to provide interventions for everyone.

*“Again capacity*,* the framework says that anyone moderately and severely frail should have a care plan in place*,* so it is about the capacity to do that. Are GPs going to do that*,* are community services going to do that…” (P13*,* Commissioner).*

*“Obviously capacity*,* although we’ve got the frailty pathways*,* could we actually do the work. The numbers that are coming through can be quite big” (P18*,* GP).*

*“You can’t eat an elephant in one go … LFF covers a third of our older population” (P11*,* Senior Pharmacist).*

#### Subtheme 2 H: Older people do not want to be labelled as ‘frail’

Several participants felt that older people do not want to be identified as frail.

*“But the actual frailty word*,* I think*,* wasn’t well known. You imagine a frail person to be very doddery*,* not able to look after themselves – people don’t like to be identified as frail” (P3*,* Senior Manager for a service).*

*“…we’ve got to be really careful about how we talk about this in our communities because people do not want to be identified as moderately frail or mildly frail” (P13*,* Commissioner).*

## Discussion

The present study has described the factors that affected the implementation of an integrated care programme for OPDFL in Luton. Studies exploring factors important to the implementation of integrated care models for older adults have typically only focused on those who are already frail or have complex conditions, without considering those in earlier stages of frailty [19, 20]. This is the first study that has explored the factors that are important for the implementation of a model of integrated care that has interventions for OPDFL, ascertaining the perspectives of both commissioners and service providers.

Two overarching themes were found, which were concerned with the strengths and limitations of existing national policies to provide integrated care for OPDFL, while the second theme was about Luton specific contextual factors that affected the implementation of the LFF. Participants viewed national initiatives, such as the formation of PCNs and the EHCH favourably, as they thought that they promoted coordination in the system. However, some service providers felt that the Government policies for older people have been reactive rather than proactive, and have focused on the prevention of admissions to hospitals and care homes, rather than of quality of life.

Among contextual factors, key leaders in the system played an important role in providing senior sponsorship for the LFF. Other studies have also suggested that leadership and common values across the health system are powerful enablers of integrated care interventions [21–23]. The availability of funding for this programme was also a facilitator. This finding is in keeping with previous work in which the non-availability of funding was identified as a barrier to the implementation of integrated care services for older people with frailty [24, 25].

Service providers also described that there were good working relationships among stakeholders, as well as a real will in the system to improve services for older people. Other studies have also found that strong working relationships, commitment, and good planning, can facilitate integrated working [26, 27]. Some GPs felt that a lack of awareness of the different frailty related interventions, and less clarity of the pathways was a reason why they did not implement them as required. This is in keeping with a French study in which providers felt that a lack of communication and awareness about the integrated care programme for older people were barriers to implementation [28]. Other studies have also reported that a lack of awareness of available services and pathways are barriers to the implementation of integrated care programmes [19, 29, 30].

At the time of the study, the Luton CCG was being merged with the other CCGs in the county. Participants were concerned that such a large-scale organisational restructuring could lead to a shift in focus from existing projects, with professionals redeployed to new roles where they would lack local knowledge. Although there are no studies on the impact of organisational restructuring on integrated care programmes for OPDFL, there has been a study in which the effectiveness of change management after organisational restructuring was evaluated. They reported that when the new special health authority was created in the NHS, employees reported that constant cycles of change with less time to stabilise lowered motivation and caused negativity towards these changes [31]. The study was conducted in 2021, and since then, major changes have occurred in the NHS. This includes the introduction of integrated care systems and integrated care boards in 2022. These local partnerships among health and care sectors aim to improve services for people [32]. The lessons learned around the challenges of structural changes and their impact on employees, as well as the barriers to delivery of existing projects, should be kept in mind for any future structural changes. The key focus should be on how to minimise disruption, take staff into confidence, offer more support, and maintain momentum in delivering existing projects.Throughout the study there were repeated comments about the COVID-19 pandemic, which was a very challenging time, and caused disruption to some of the progress made in terms of the implementation of the LFF. Although there is little known about the impact of the pandemic on integrated care programmes for OPDFL, some literature suggests that COVID-19 disrupted healthcare services and caused distress to both healthcare staff and older patients, who feared catching the virus, while the COVID-19 also created many other challenges such as pressures on the critical care capacity and mass testing of COVID-19 among healthcare professionals [33–36].

One of the issues that was raised concerned capacity, with participants feeling that the eFI identifies a huge proportion of the older population as moderate or severely frail. However, they also felt that the system lacks the capacity, in both resources and workforce, to implement the care pathways for all moderately or severely frail older people. Likewise, in a study of 16 integrated care pilots in England, participants felt that the intervention was too big and too complex [24]. They also identified that substantial time and resources should be dedicated to the implementation, in order for the expected improvements to occur. Other studies have also found capacity issues, such as a lack of time and resources, as barriers to integrated care delivery [26, 29].

A negative connotation of being identified as frail was also reported, which is in keeping with other studies in which the beliefs and knowledge of older people about frailty have been explored [28, 37].

This study aimed at exploring the perceptions of both commissioners and service providers. However, it was not possible to recruit service providers from acute care services, except for one geriatrician. Nevertheless, it was possible to recruit participants who had different roles within the LFF, which meant that in-depth views about the factors that are crucial for the implementation of an integrated care programme for OPDFL were able to be collected.

## Conclusion

This study was conducted to explore factors that affect the implementation of an integrated care programme in Luton. National initiatives that facilitate coordination are helpful, however, a traditional reactive approach towards older people who are frail is counterproductive. Identifying and managing older people with moderate and severe frailty can only be achieved if the system is provided with more resources. While the term frailty might help service providers to identify people, it needs to be used carefully, as patients can view it negatively. This study also found contextual factors that affect the implementation of integrated care, such as having key leaders, while the availability of funding and good working relationships are also important. A lack of system level awareness of care pathways could also lead to variation in their implementation, while implementation the LFF during the COVID-19 pandemic proved to be especially challenging.

## Data Availability

The data used and analysed during the current study are available from the corresponding author on reasonable request.

## Abbreviations

CCG: Clinical Commissioning Group
GPs: General Practitioners
LFF: Luton framework for frailty
OPDFL: Older people with different frailty levels
